# Supplementary material for: Numerical Analysis of the Light Modulation by the Frustule of Gomphonema parvulum: The Role of Integrated Optical Components
Source: Nanomaterials (Basel). 2022 Dec 26;13(1):113. doi: 10.3390/nano13010113 (PMC9823621; doi:10.3390/nano13010113)
Supplement: Supplementary file 1 [file nanomaterials-13-00113-s001.zip › nanomaterials-2119041-supplementary.pdf]

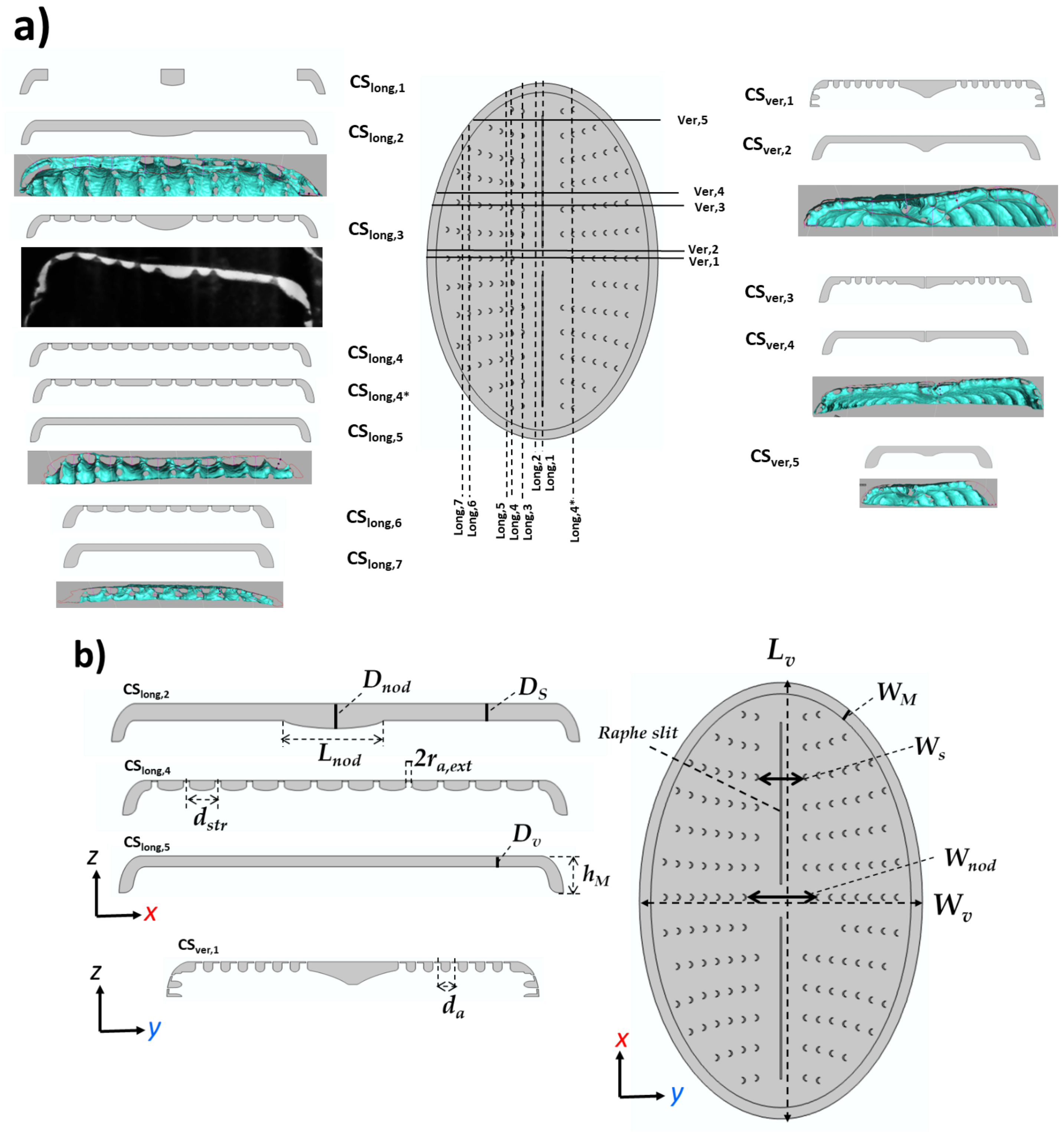

Figure S1. (a) the selected vertical & longitudinal CSs across the statistically representative 3D valve model that is shown in the center (at the external top view). The five vertical CSs and the eight longitudinal CSs schemed at the right and left, respectively, along with some 2D cross-sections from the 3D reconstructed data to show comparable places in the valve, reflecting the similarity between the final 2D CSs and the actual structure. (b) the important structural parameters mentioned in Table 1.

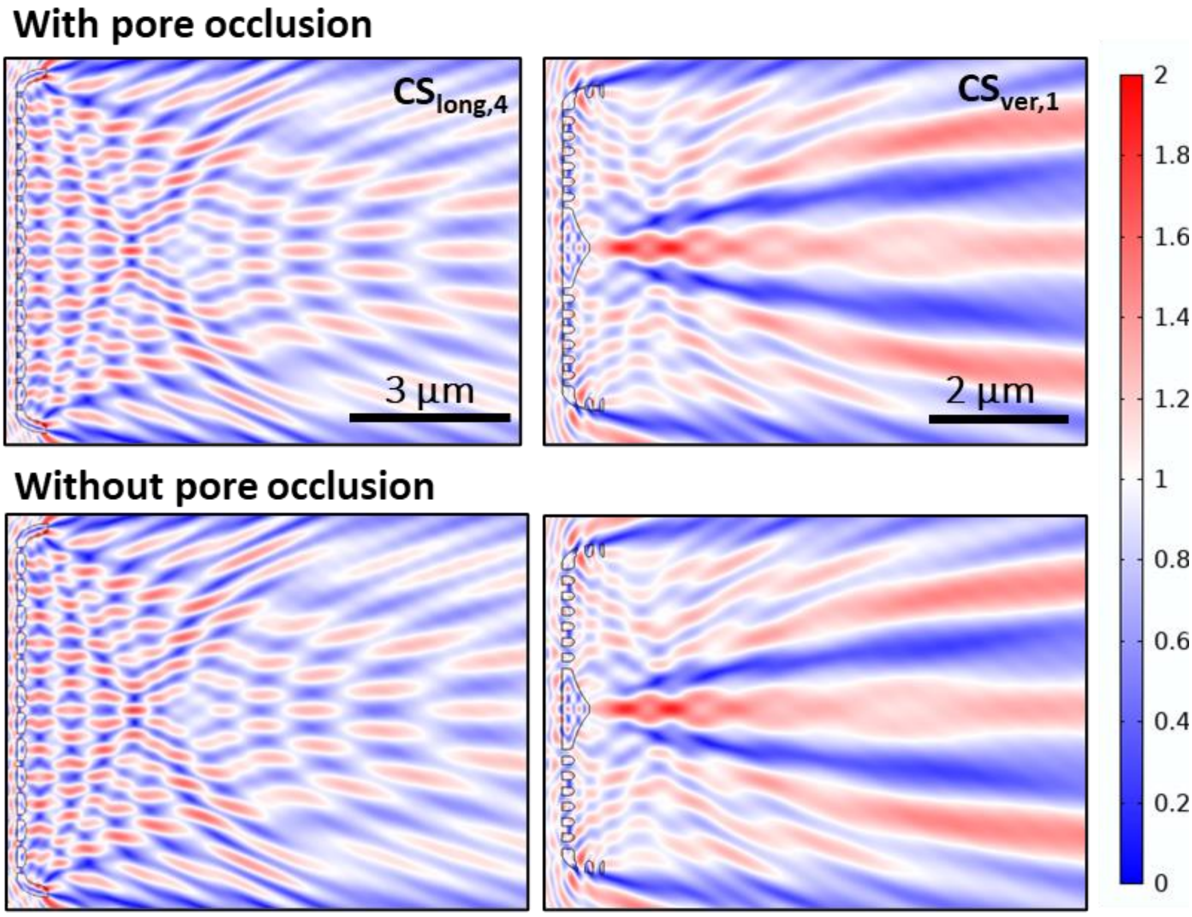

Figure S2. The effect of pore occlusions on interference pattern of $CS_{long,4}$ and $CS_{ver,1}$ at λ_{vacc}= 350 nm.

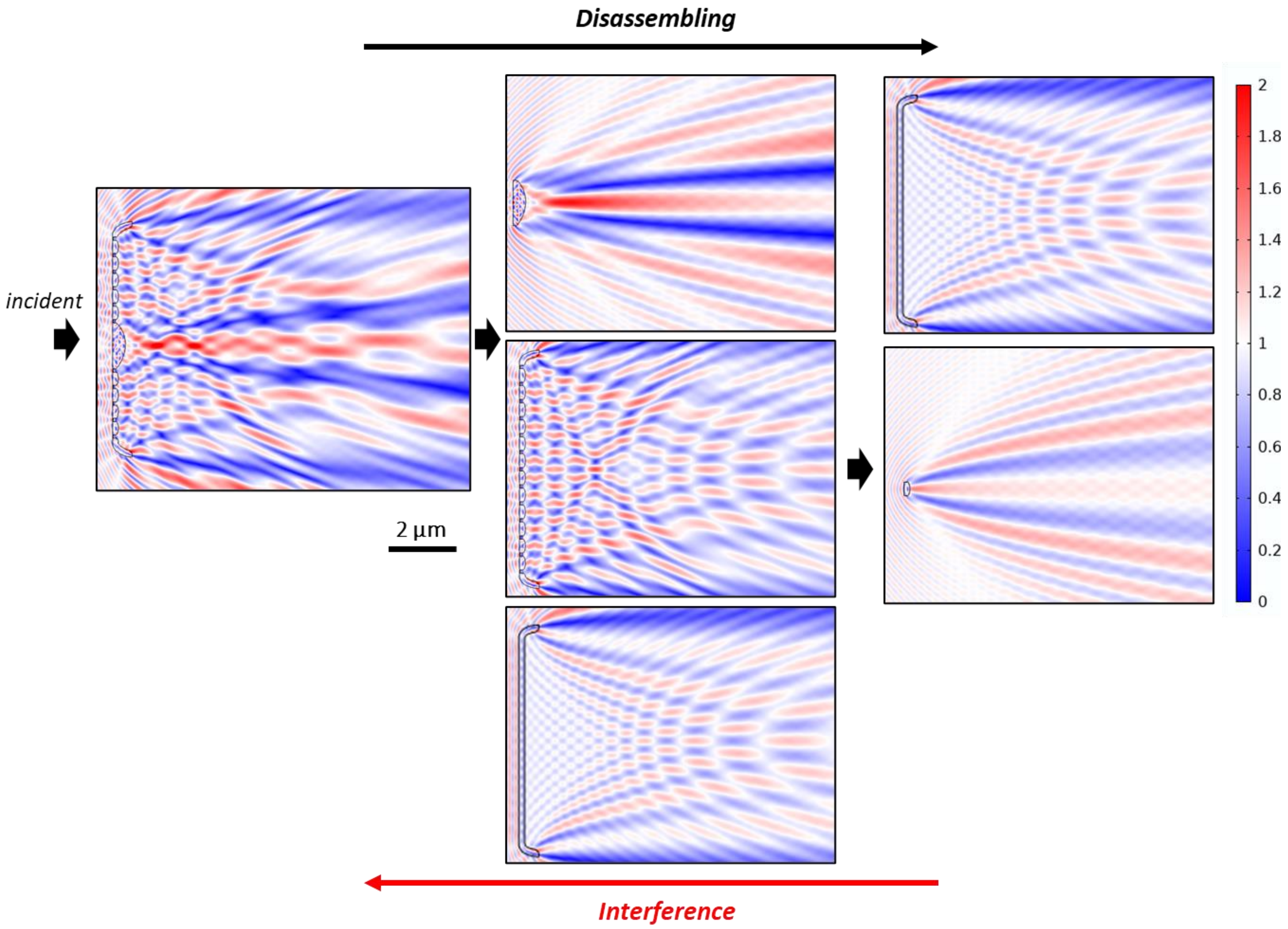

Figure S3. A schematic diagram shows the complex interference pattern observed in $CS_{long,4}$ at λ_{vacc}= 350 nm, while disassembling its distinct structural components shows the contribution of each element to the interference pattern.

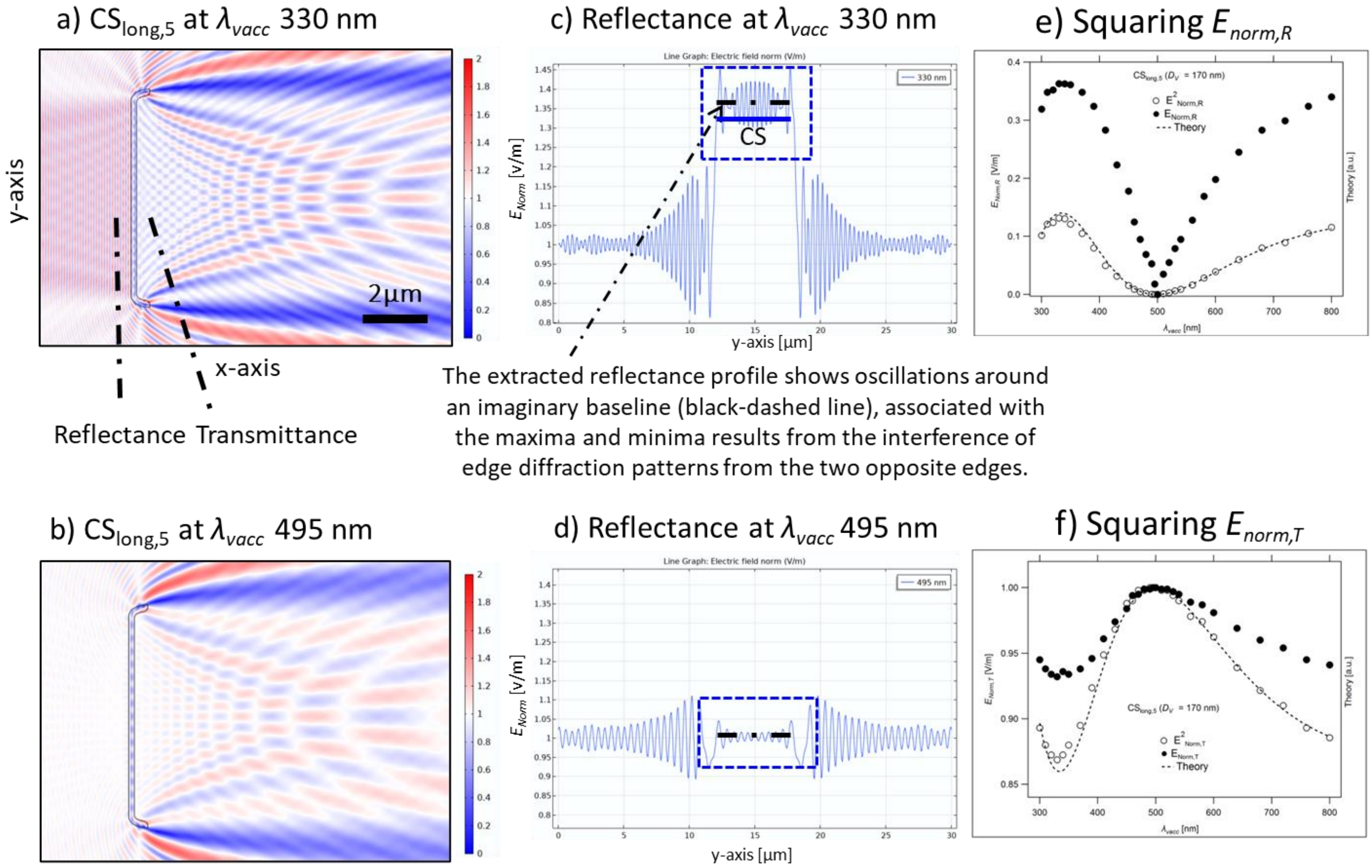

Figure S4. An example shows the interference pattern of $CS_{long,5}$ at λ_{vacc} 330 (a) and 495 (b), besides the extraction method of the reflectance $E_{Norm,R}$ that shows a constructive interference maximum (c) and minimum (d). The reflectance and transmittance should produce a flat wavefront, confirmed through the simulation of an extended thin slab (of L_{sl} 80 µm in a larger simulation box). Therefore, the oscillations that appear in the extracted reflectance in (c and d) were associated with edge diffraction. Thus, to read out the CSs' reflectance and transmittance with the presence of overlayed edge diffraction, we estimated the position of the baseline (associated with the expected flat wavefront) as an average between the maxima and minima in the observed oscillations (dashed black line in c and d). The error in the measurements was estimated by averaging the extracted E_{Norm} from three consecutive x-lines close to the CS. Squaring the E_{Norm} - after the subtraction of E_{input} from the extracted E_{Norm} only in the case of reflectance - gave the best match to theoretical calculations (e and f).

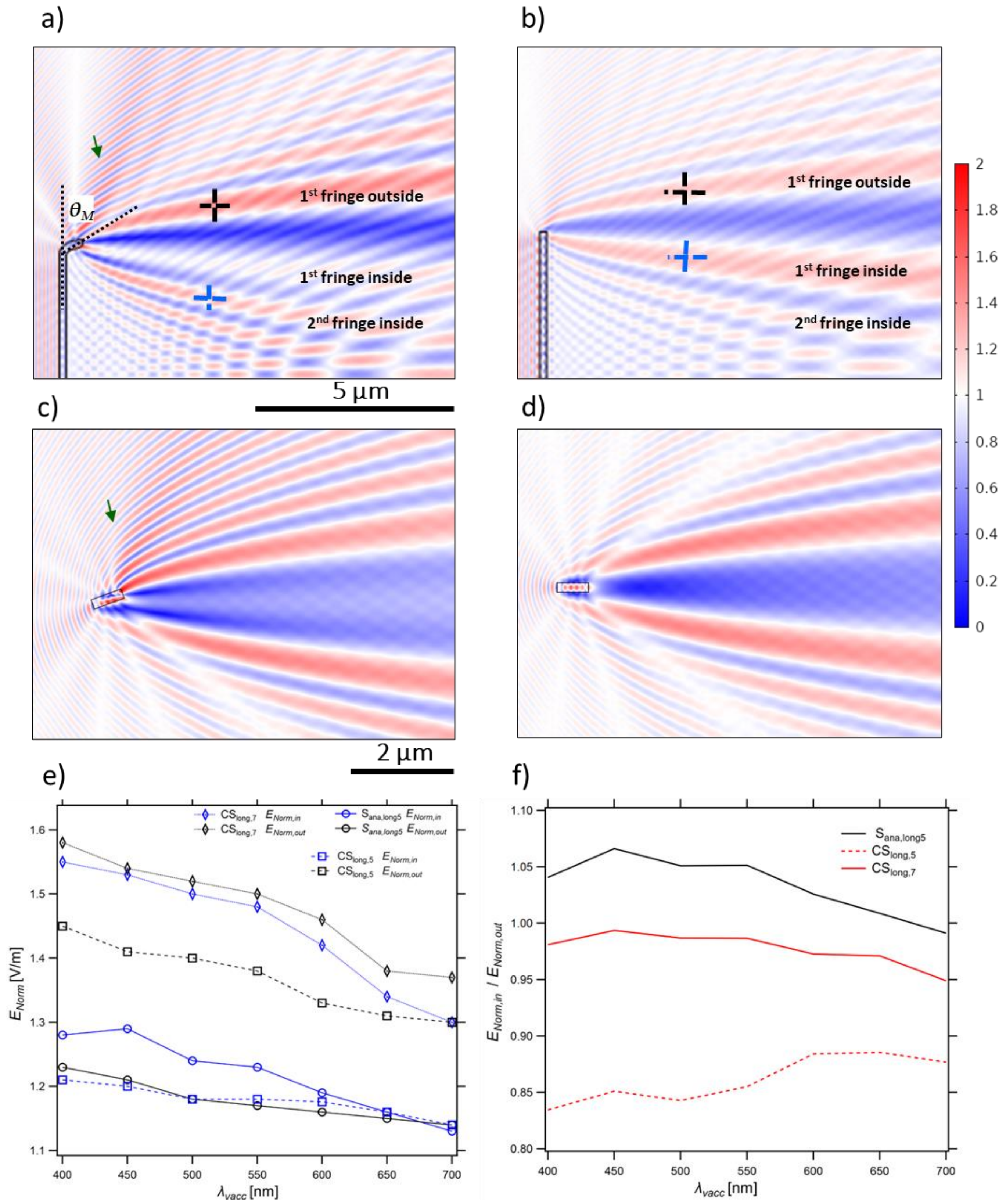

Figure S5. The edge diffraction fringes in $CS_{long,5}$ (a) and $S_{ana,long5}$ (b) at λ_{vacc} 300 nm in the air. Tilted $S_{ana,M}$ in (c) compared to in-plane alignment in (d) at λ_{vacc} 300 nm in air. The plot in (e) shows λ_{vacc} dependency of the edge diffraction fringes strength as well as the changes in the $E_{Norm,in}$ and $E_{Norm,out}$ for $CS_{long,5}$, $CS_{long,7}$, and $S_{ana,long5}$. While the plot in (f) shows the difference in $E_{Norm,in}/E_{Norm,out}$ ratios. By adding the tilted mantle to the thin slab element in $CS_{long,5}$, the strength of the outside and inside fringes seem to be enhanced, except the 1st fringe inside is diminished. The Crosses in (a) and (b) represent the x-y-positions considered for E_{Norm} extraction that are plotted in (e). The error was up to ± 0.07, ± 0.05, or ± 0.03 V/m in the case of $CS_{long,5}$, $CS_{long,7}$, or $S_{ana,long5}$, respectively. The error in the measurements was estimated by averaging the extracted E_{Norm} from three consecutive points at the same fringe. The ratio in (f) was calculated based on the average values.

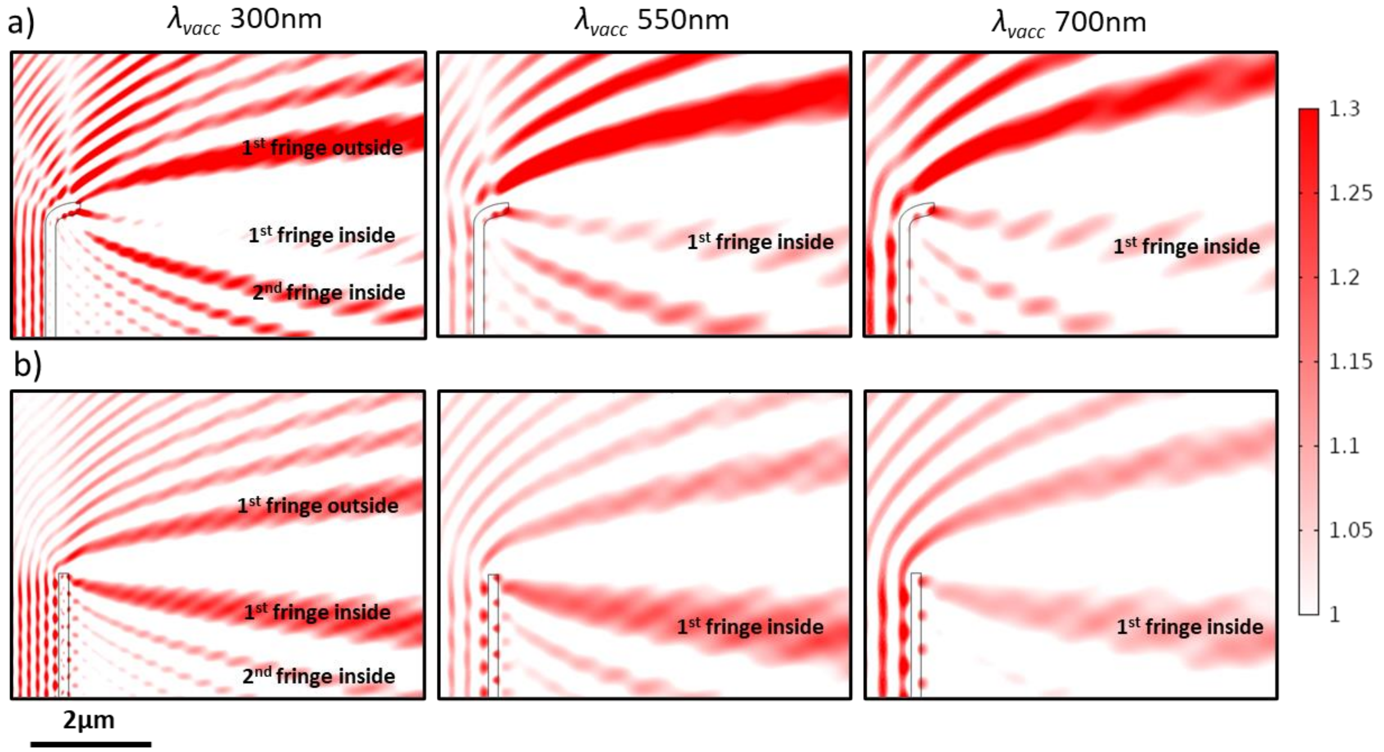

Figure S6. The λ_{vacc} dependency of the edge diffraction fringes in $CS_{long,5}$ (a) and $S_{ana,long5}$ (b) in the air. The color code emphasizes the E_{Norm} enhancement in red ($E_{Norm} > 1$ V/m) while keeping both the E_{input} and the E_{Norm} reduction in white ($E_{Norm} \leq 1$ V/m), not emphasized. The presence of the mantle in (a) also spatially delays the 1st and 2nd diffraction fringes inside.

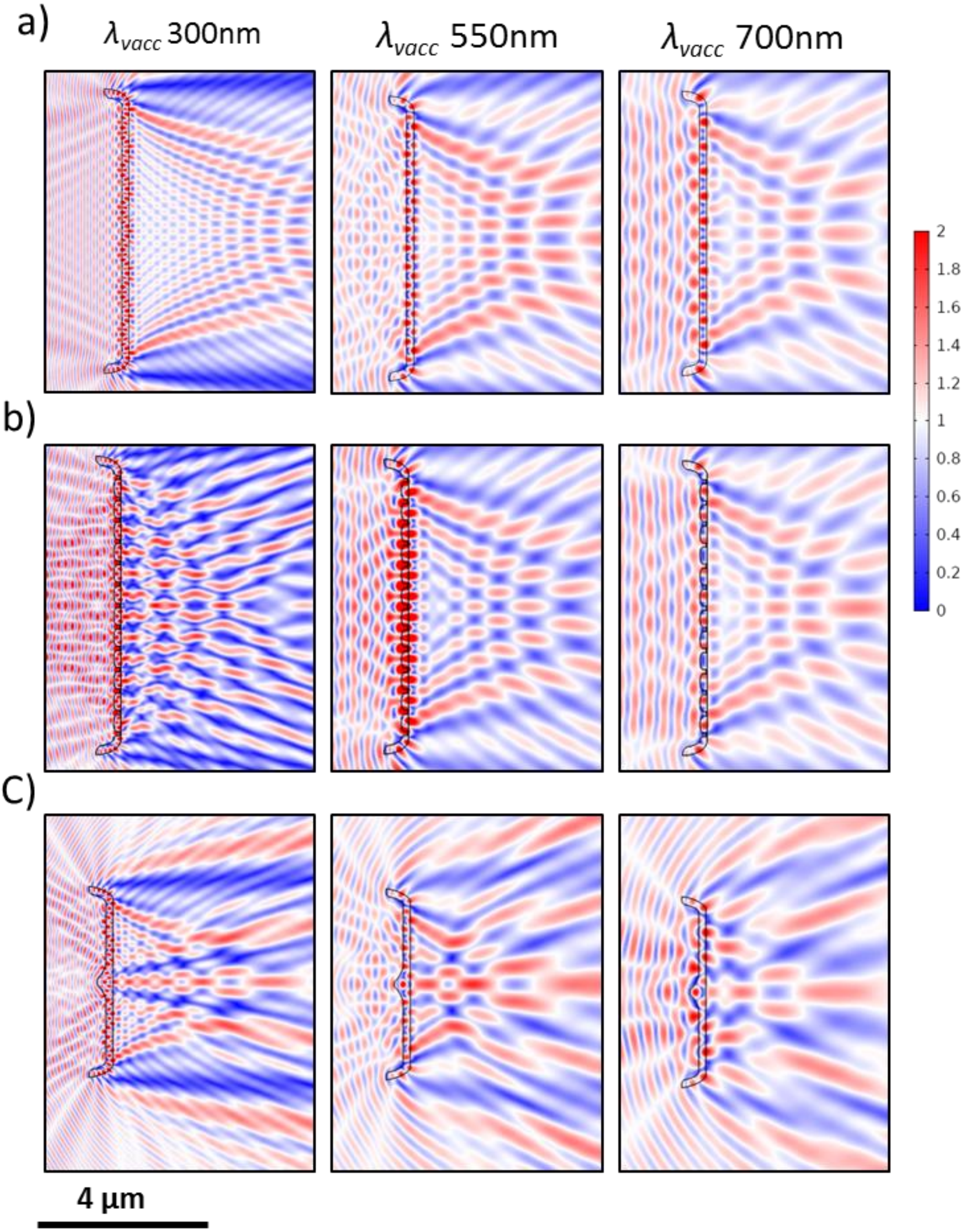

Figure S7. The mantle-coupled GMR-like behavior appears in $CS_{long,5}$ (a), $CS_{long,4}$ (b), and $CS_{ver,2}$ (c) in the air.

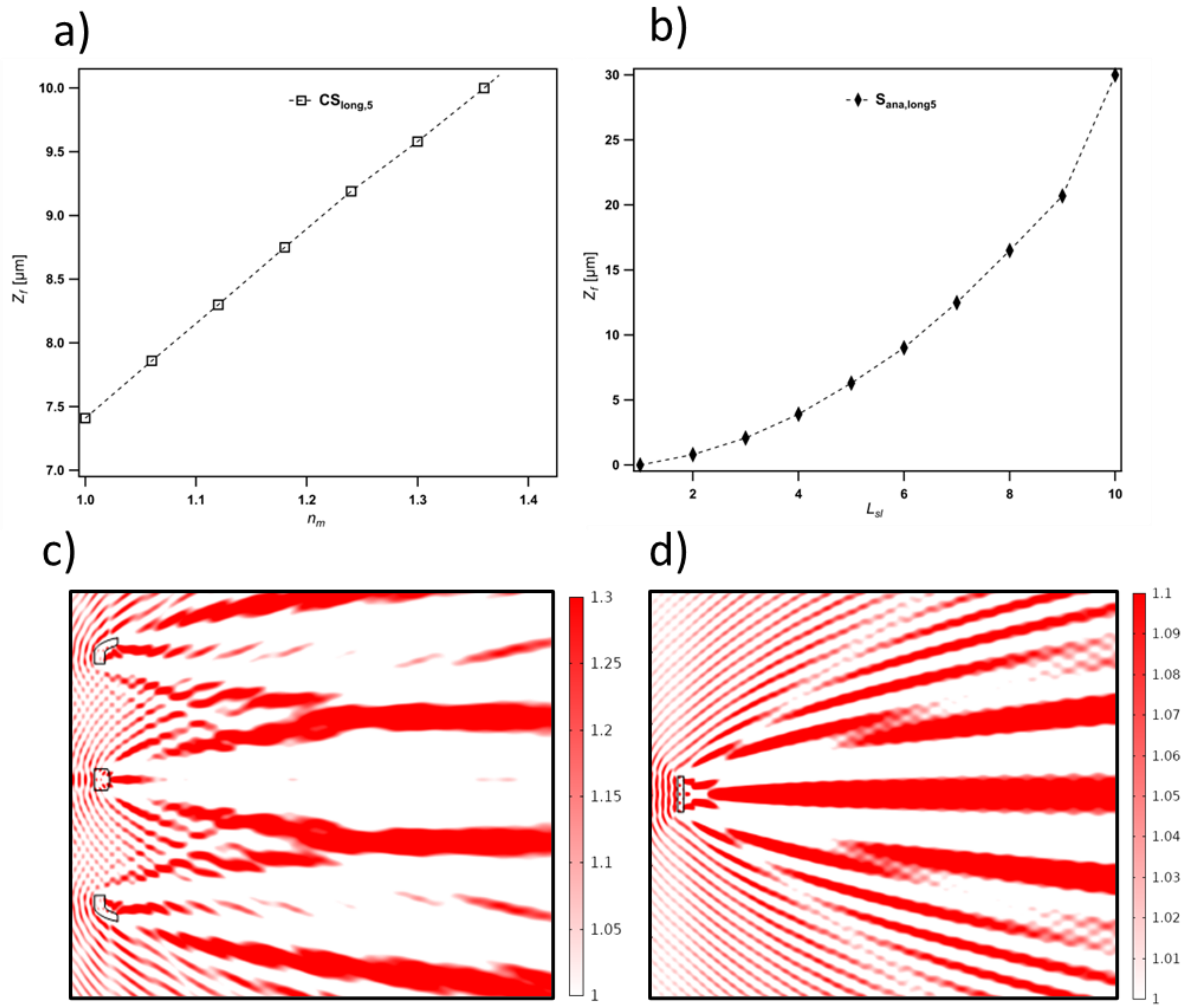

Figure S8. (a) The n_m dependency of Z_f of CS$_{long,5}$ at n_v = 1.46 and λ_{vacc} 330 nm. (b) The L_{sl} dependency of Z_f of S$_{ana,long5}$ in air at λ_{vacc} 330 nm. (c) the interference pattern of CS$_{long,1}$ at λ_{vacc} 300 nm. (d) PJ generated by Sana,long5 of L_{sl} = 1 µm at 330 nm in air.

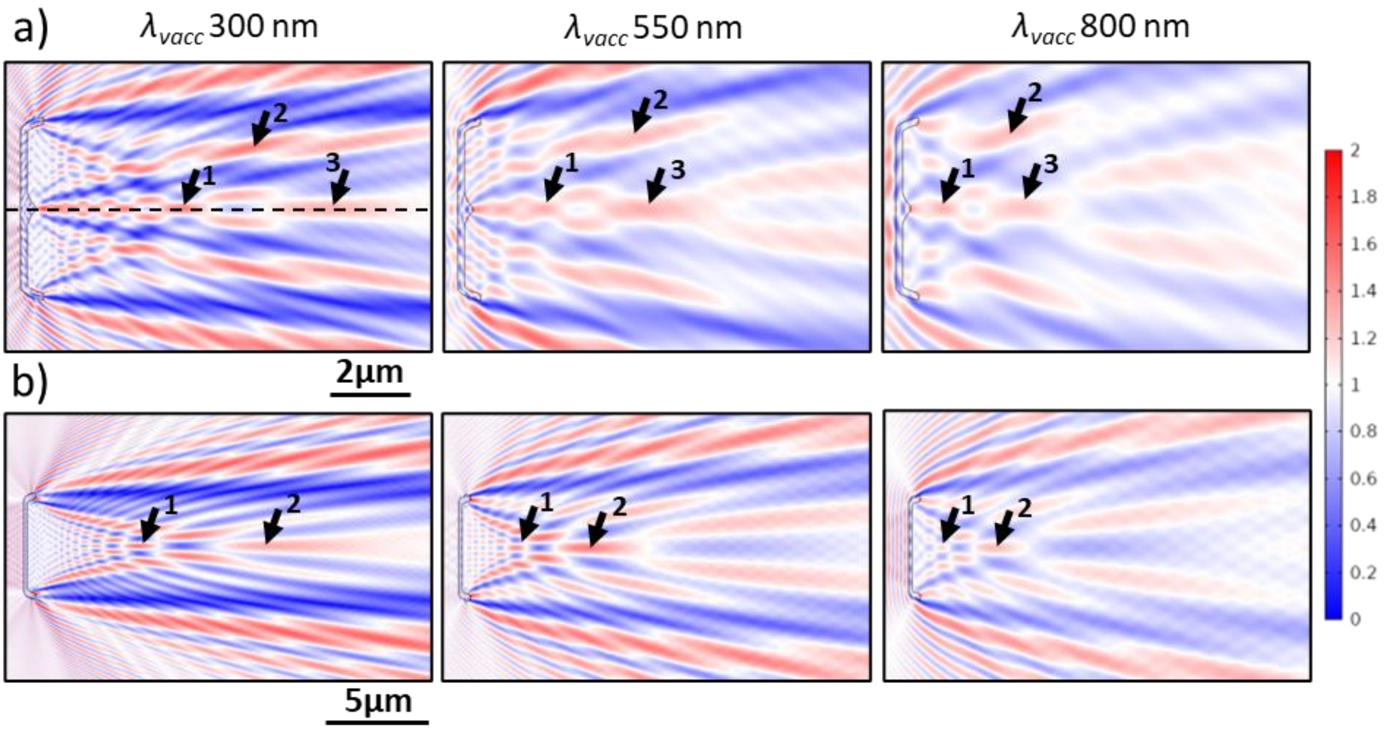

Figure S9. Tracking different focusing spots while increasing λ_{vacc} in $CS_{ver,2}$ (a) and $CS_{long,7}$ (b) in air.

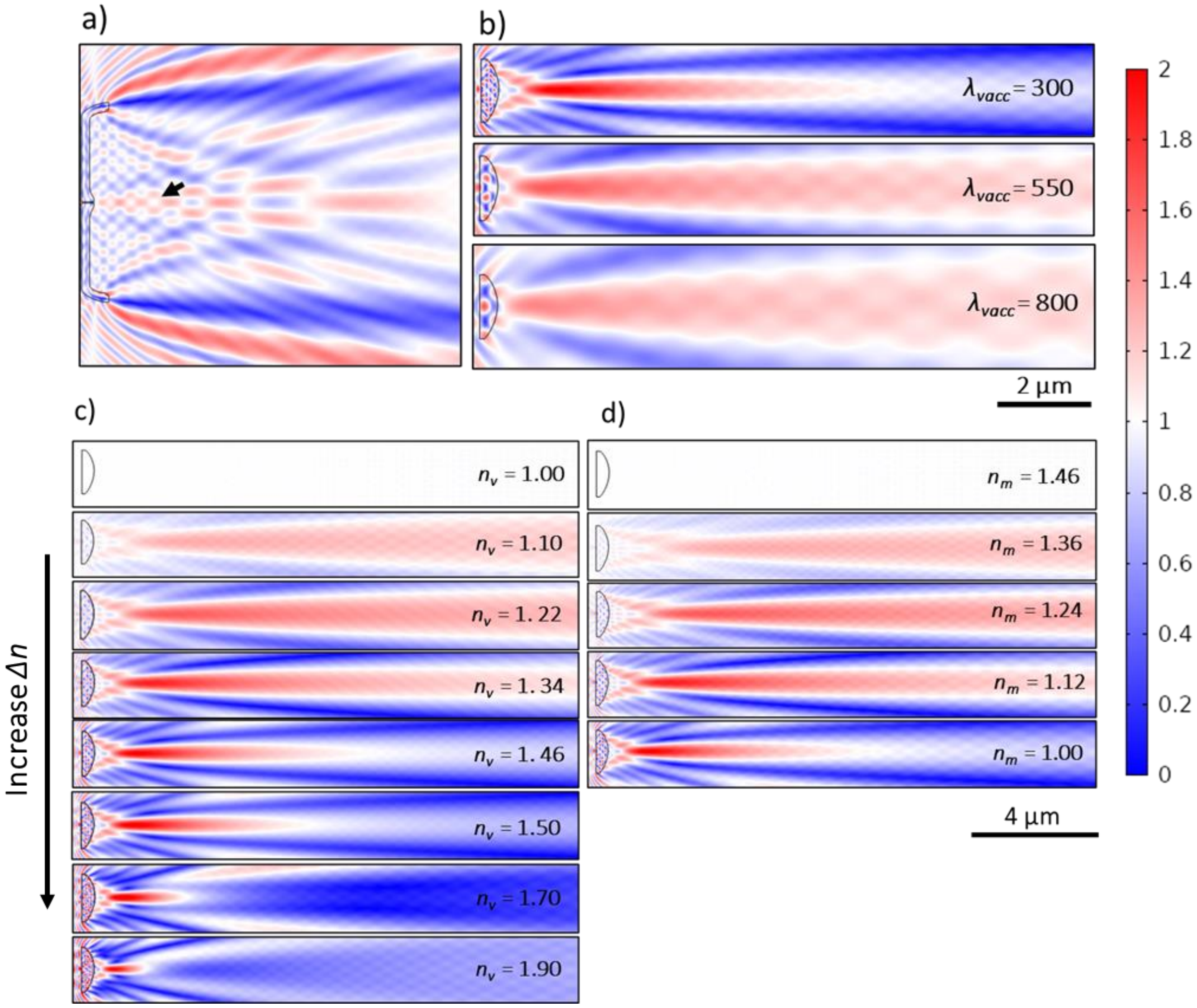

Figure S10. Photonic jet generation in $CS_{ver,4}$ at λ_{vacc} 350 nm (a), the λ_{vacc} dependency of the generated PJ from the disassembled lens-like structure ($CS_{long,3\ /nodule}$) in the air (b), the Δn dependency of the generated PJ from $CS_{long,3\ /nodule}$ at λ_{vacc} 300 nm with changing n_v for n_m = 1 (c) or changing n_m for n_v = 1.46 (d). In both (c) and (d), the images were arranged to show Δn increasing toward below.

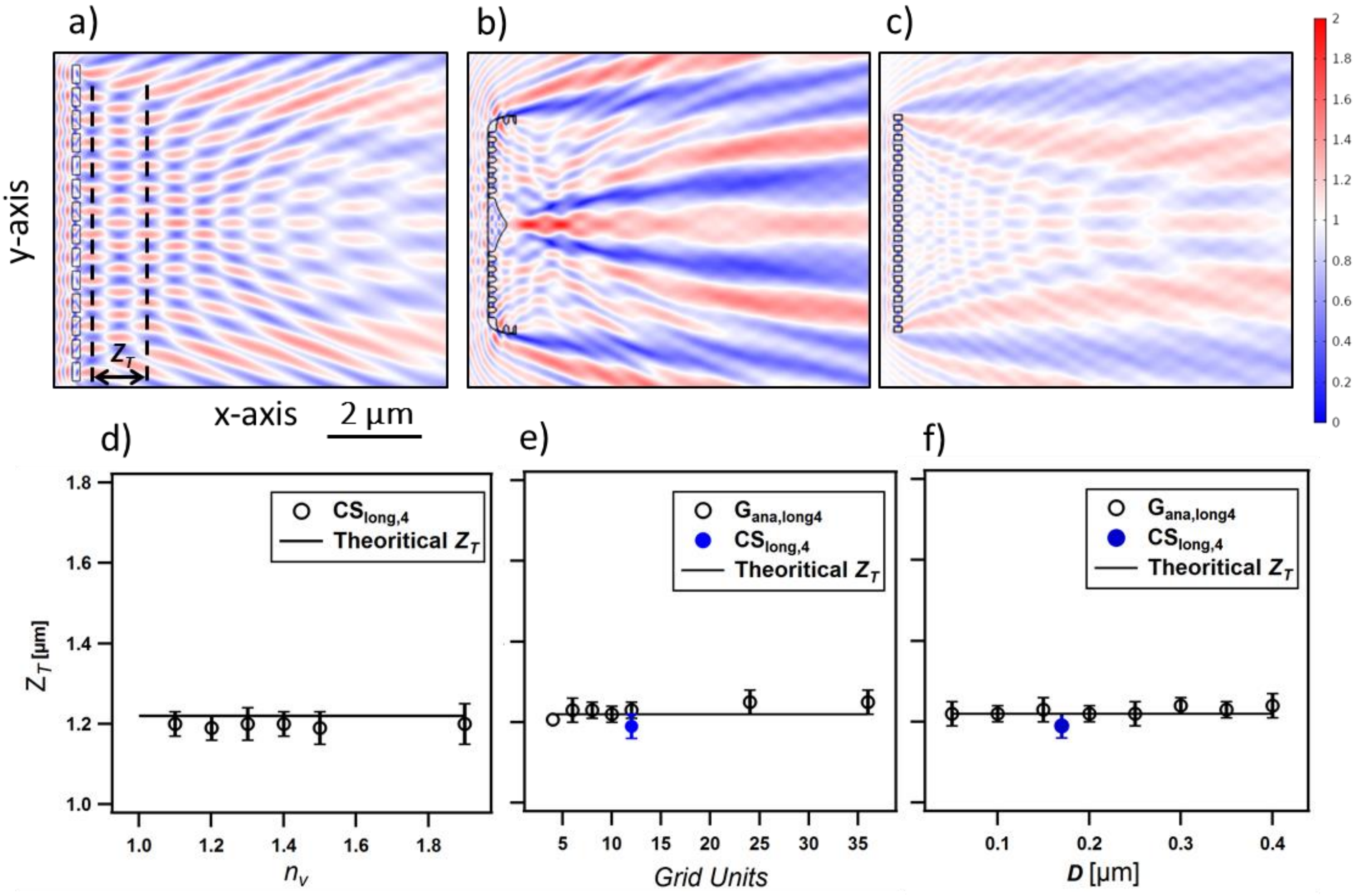

Figure S11. The analytical grid $G_{ana,long4}$ equivalent to $CS_{long,4}$ (a), $CS_{ver,1}$ (b), and the analytical grid $G_{ana,ver1}$ equivalent to $CS_{ver,1}$ (c) at λ_{vacc}= 350 nm in the air. The three graphs show the Z_T independency of the changes in n_v (n_m =1.00) of $CS_{long,4}$ (d), the grid units number of $G_{ana,long4}$ (e), or its thickness (f) at λ_{vacc}= 350 nm in the air compared to the theoretical expectation. The error bars in graphs represent the uncertainty in the measured Z_T due to deformation of the fringes.

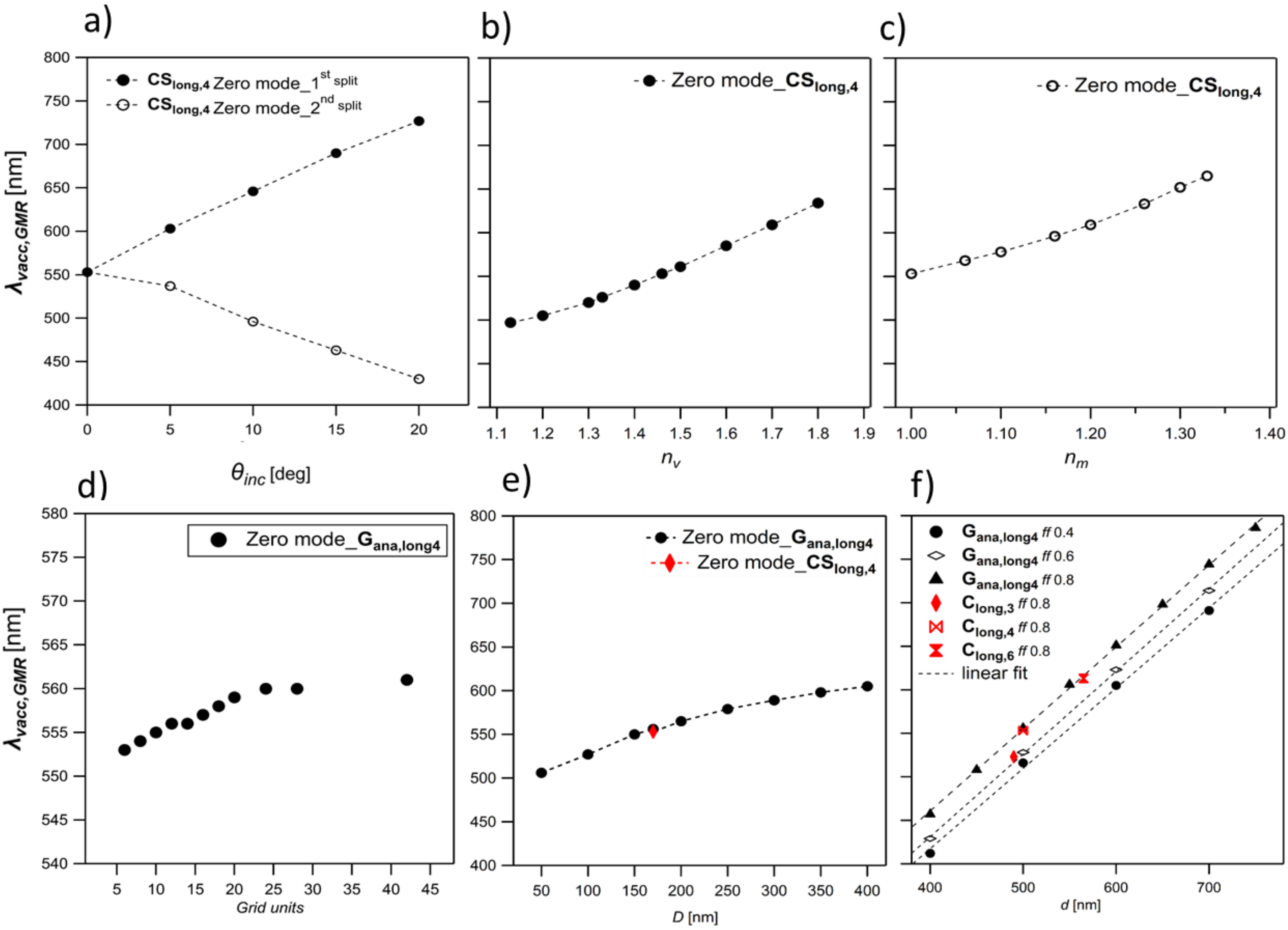

Figure S12. The graphs show CS$_{long,4}$ $\lambda_{vacc,GMR}$-dependency of zero mode on θ_{inc} (a), changing n_v (n$_m$ =1.00) (b), and changing n_m (n$_v$ =1.46) (c). While graphs in (d), (e), and (f) show $\lambda_{vacc,GMR}$-dependency of zero mode on changing grid units, *D*, or *d* (at different *ff*), respectively, of G$_{ana,long4}$ in air.

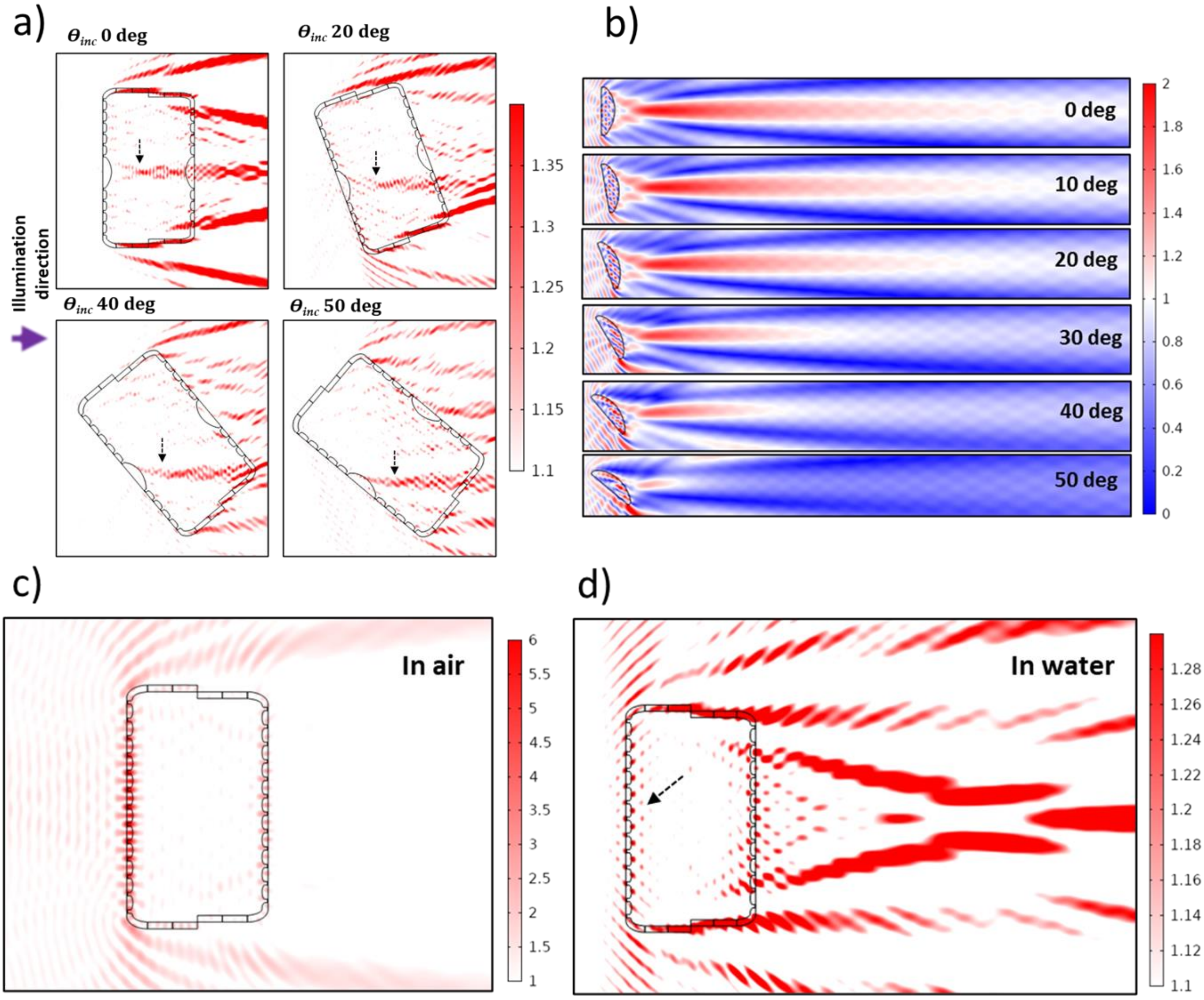

Figure S13. (a) the θ_{inc}-dependency of the PJ generated by the nodule inside $CS_{long3,frustule}$ at λ_{vacc} 400 nm in water. The color code starts from 1.1 V/m to emphasize the PJ from the talbot fringes. (b) the θ_{inc}-dependency of the PJ generated by the dissembled nodule at λ_{vacc} 350 nm in air. (c) zero mode grid-coupled GMR in $CS_{long4,frustule}$ at $\lambda_{vacc,GMR}$ 553 nm in air and (d) at $\lambda_{vacc,GMR}$ 665 nm in water. The black arrow in (d) indicates what seems as an increase in the evanescence field penetration.
